# Supplementary material for: Disease-modifying effects of natural Δ9-tetrahydrocannabinol in endometriosis-associated pain
Source: eLife. 2020 Jan 14;9:e50356. doi: 10.7554/eLife.50356 (PMC6977967; doi:10.7554/eLife.50356)
Supplement: Transparent reporting form [file elife-50356-transrepform.docx]

***eLife’s* transparent reporting form**

We encourage authors to provide detailed information *within their submission* to facilitate the interpretation and replication of experiments. Authors can upload supporting documentation to indicate the use of appropriate reporting guidelines for health-related research (see [EQUATOR Network](http://www.equator-network.org/%20)), life science research (see the [BioSharing Information Resource](https://biosharing.org/)), or the [ARRIVE guidelines](http://www.plosbiology.org/article/info:doi/10.1371/journal.pbio.1000412) for reporting work involving animal research. Where applicable, authors should refer to any relevant reporting standards documents in this form.

If you have any questions, please consult our Journal Policies and/or contact us: [editorial@elifesciences.org](mailto:editorial@elifesciences.org).

**Sample-size estimation**

- You should state whether an appropriate sample size was computed when the study was being designed
- You should state the statistical method of sample size computation and any required assumptions
- If no explicit power analysis was used, you should describe how you decided what sample (replicate) size (number) to use

Please outline where this information can be found within the submission (e.g., sections or figure legends), or explain why this information doesn’t apply to your submission:

Sample size was based on previous studies in our laboratory using comparable behavioral approaches (La Porta et al., Pain 2015; Busquets-García et al., Nat Med. 2013; La Porta et al., Pain 2013). Subsequent power analysis was conducted for each test and is provided in the respective Source data files.

**Replicates**

- You should report how often each experiment was performed
- You should include a definition of biological versus technical replication
- The data obtained should be provided and sufficient information should be provided to indicate the number of independent biological and/or technical replicates
- If you encountered any outliers, you should describe how these were handled
- Criteria for exclusion/inclusion of data should be clearly stated
- High-throughput sequence data should be uploaded before submission, with a private link for reviewers provided (these are available from both GEO and ArrayExpress)

Please outline where this information can be found within the submission (e.g., sections or figure legends), or explain why this information doesn’t apply to your submission:

The first, second, and third experiments assessing the effects of the ectopic endometrium, and the acute antinociceptive effects of THC were performed once. The fourth experiment assessing the effect of repeated THC exposure contained groups treated with vehicle representing the conditions of the first experiment. This fourth experiment was performed twice, and data were pooled and analyzed together. The fifth experiment assessing tolerance development after repeated administration of THC was performed once.

Number of biological replicates (mice or tissue samples) and technical replicates (number of sections for immunohistochemistry, and triplicates for ELISA) are specified in the Materials and Methods section. Detailed raw data for each biological replicate is included in the respective Source data file.

No outliers were excluded for the statistical analysis.

**Statistical reporting**

- Statistical analysis methods should be described and justified
- Raw data should be presented in figures whenever informative to do so (typically when N per group is less than 10)
- For each experiment, you should identify the statistical tests used, exact values of N, definitions of center, methods of multiple test correction, and dispersion and precision measures (e.g., mean, median, SD, SEM, confidence intervals; and, for the major substantive results, a measure of effect size (e.g., Pearson's r, Cohen's d)
- Report exact p-values wherever possible alongside the summary statistics and 95% confidence intervals. These should be reported for all key questions and not only when the p-value is less than 0.05.

Please outline where this information can be found within the submission (e.g., sections or figure legends), or explain why this information doesn’t apply to your submission:

Details of the statistical tests used are provided in the Statistics subsection of the Materials and Methods section.

Mean, SEM and significance level are shown, together with the individual data points whenever possible. Figure legends specify the statistical tests used and the levels of significance. Source data files contain raw numerical data for each data point in the figures, mean, SD, SEM, confidence intervals, exact values of N, exact p-values, results of Bonferroni correction when appropriate, and measures of power and effect size.

(For large datasets, or papers with a very large number of statistical tests, you may upload a single table file with tests, Ns, etc., with reference to sections in the manuscript.)

**Group allocation**

- Indicate how samples were allocated into experimental groups (in the case of clinical studies, please specify allocation to treatment method); if randomization was used, please also state if restricted randomization was applied
- Indicate if masking was used during group allocation, data collection and/or data analysis

Please outline where this information can be found within the submission (e.g., sections or figure legends), or explain why this information doesn’t apply to your submission:

Treatments were randomized among groups, and experiments and measurements were performed blinded for pharmacological and surgical conditions, as stated in the Materials and methods section.

**Additional data files (“source data”)**

- We encourage you to upload relevant additional data files, such as numerical data that are represented as a graph in a figure, or as a summary table
- Where provided, these should be in the most useful format, and they can be uploaded as “Source data” files linked to a main figure or table
- Include model definition files including the full list of parameters used
- Include code used for data analysis (e.g., R, MatLab)
- Avoid stating that data files are “available upon request”

Please indicate the figures or tables for which source data files have been provided:

Source Data files for each figure contain all the raw data and statistical analyses conducted with the IBM SPSS 23 software (Chicago, IL, USA). Data availability statement is included at the end of the manuscript. In addition, the ARRIVE guidelines have been followed and the ARRIVE checklist is provided as supporting documentation.
